# Ultrasound in the diagnosis of pneumothorax: a survey of current practice

**DOI:** 10.1186/cc13446

**Published:** 2014-03-17

**Authors:** T Berlet, T Fehr, T Merz

**Affiliations:** 1 Inselspital/University Hospital Bern, Switzerland

## Introduction

The purpose of this study was to survey current practice for the use of lung ultrasound (LUS) in the diagnosis of pneumothorax.

## Methods

Physician sonographers accredited by the German Medical Ultrasound Society (DEGUM) for ultrasonography in surgery, anaesthesia or medicine were invited to participate in an online survey. Frequency of exposure to patients with suspected pneumothorax, frequency of LUS use, assessment of diagnostic accuracy of LUS for ruling-out or ruling-in pneumothorax and preferences regarding technical aspects were enquired about.

## Results

**Figure 1:**
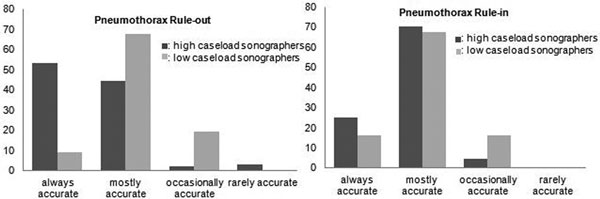
**Accuracy of LUS in pneumothorax**.

Eighty-nine physicians responded. Average exposure to pneumothorax cases was 1/week. Fifty-five per cent of respondents used LUS 'always' or 'frequently'. Thirty-four per cent of physicians rated LUS as 'always accurate', and a further 54% as 'accurate in a majority of cases' in ruling out pneumothorax. Twenty-one per cent rated LUS as 'always accurate' and 69% as 'accurate in a majority of cases' in ruling in pneumothorax. Physicians reporting frequent exposure to pneumothorax patients used LUS in a higher proportion of cases ('high caseload sonographers') and were more confident to rule out pneumothorax (Figure 1). In total, 16 different combinations of transducers, probe orientations and scanning modes were reported. Linear transducers, sagittal probe-orientation, and B-mode and M-mode scanning were most often selected (38%).

## Conclusion

Physicians' use of LUS in the diagnosis of pneumothorax was modest. Assessment of diagnostic accuracy gave markedly lower scores than reported in clinical trials [1]. Correlation between frequency of exposure, likelihood of LUS usage and confidence in diagnostic accuracy warrants further research into the nature of the learning curve. Considerable variations regarding technical aspects of LUS reflect the ambiguity of current recommendations [2]. More research is required to establish the most efficient way of performing LUS for suspected pneumothorax and efforts need to be made to promote its use in these scenarios.
